# Intracerebroventricular dopamine for Parkinson's disease

**DOI:** 10.18632/oncotarget.17596

**Published:** 2017-05-03

**Authors:** David Devos, Jean-Christophe Devedjian, Caroline Moreau

**Affiliations:** Department of Medical Pharmacology, Lille University de Lille, CHU of Lille, INSERM UMRS-1171, Faculty of Medecine of Lille, Lille, France

**Keywords:** Parkinson's disease, dopamine, L-dopa related motor complications, treatment, dyskinesia

Parkinson's disease (PD) is the second most frequent neurodegenerative disorder worldwide. The depletion of dopamine in the nigro-striatal pathway is a main pathological hallmark that requires continuous and focal restoration. Since dopamine does not cross the digestive mucosa or the blood brain barrier, its lipophilic precursor L-dopa has been employed and remains the pivotal oral medication. However, after 5-7 years of L-dopa, many pharmacokinetic drawbacks contribute to the occurrence of severe motor and non-motor complications (i.e. under and overdoses) [1]. Indeed L-dopa has a short half-life, limited and variable reabsorption through the digestive and blood brain barriers and potentially harmful peripheral distribution. Moreover, L-dopa requires the aromatic L-amino acid decarboxylase for the synthesis of dopamine, which declines in the striatum with disease progression [2].

A viable optimal therapeutic regime would be to continuously compensate the deficit in dopamine in order to prevent oscillations in neurotransmitter concentration [5]. A continuous intracerebroventricular (i.c.v.) administration better mimics the physiological released of dopamine caused by the tonic background activity of dopaminergic neurons. The i.c.v administration of dopamine can be performed through an abdominal pump with subcutaneous catheter from the pump to the lateral ventricle. The latter is directly upstream to the third ventricle, being very close to the bilateral striatum (Figure).

**Figure 1:**
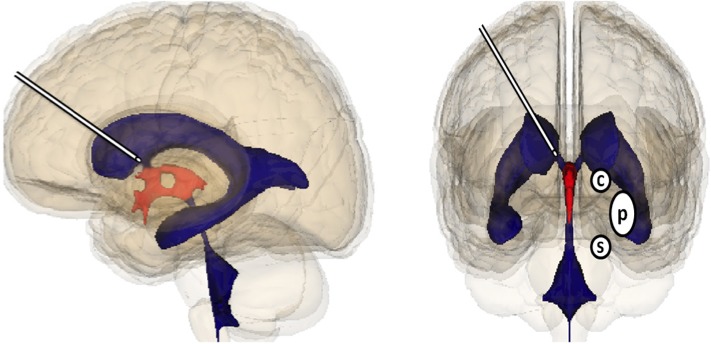
Concept of continuous intracerebroventricular administration of anaerobic dopamine for Parkinson's disease The cannula is represented in the lateral ventricle close to the entrance of the third ventricle. Striatum involves caudate nucleus (C) putamen (P), S means substantia nigra.

De Yebenes et al. [4] previously demonstrated that i.c.v. administered dopamine with an anti-oxidant adjuvant (sodium metabisulfite; SMBS) transiently improved motor handicap and increased dopamine in rat brains with unilateral neurotoxin 6-hydroxydopamine (6-OHDA)-induced damage as well as 1-methyl-4-phenyl-1,2,3,6-tetrahydropyridine (MPTP) intoxicated monkeys. The clinical feasibility of this administrative route has been supported by two PD patient case reports of dopamine infusion to the frontal ventricle, whereby a reduction in motor handicap was observed [6, 8]. The first human case report [8] described a good tolerance to dopamine infusion over 1 year. A subsequent human case report [6] showed that long-term i.c.v. dopamine provides a smooth control of motor symptoms.

However, both preclinical and clinical reports also highlight two overriding problems that prevented further development; (i) occurrence of tachyphylaxia and (ii) oxidation of dopamine causing enhanced dopamine metabolism and oxidative stress. Indeed, PD patients from previous studies received dopamine prepared aerobically (O-dopamine) and at the same dose throughout a 24 hours cycle. Prior experience obtained from the use of an apomorphine pump and duodopa® has identified the need to differentiate between diurnal and nocturnal minimum efficient dose in order to avoid worsening motor fluctuations [3].

Recently, the authors have overcome these challenges [7]. Indeed, deleterious effect of dopamine oxidation was avoided by anaerobic preparation of dopamine (A-dopamine: prepared, stored and administered in very low oxygen conditions). A-dopamine did not need a conservator (sodium metabisulfite, SMBS), which has detrimental effects. A-dopamine restored motor function (i.e. actimetry analysis) of the mouse mice, 7 days after MPTP insult, and had a broader therapeutic index than peripheral L-dopa treatment. A-dopamine demonstrated good diffusion into the striatum. Concomitantly, A-dopamine induced a dose dependent increase of nigro-striatal dopaminergic neurons that was not evident with either O-dopamine or L-dopa. A-dopamine and L-dopa treatments were equally protective in regard to the redox state of dopaminergic neurons.

In addition, greater advances in programmable pumps now minimize tachyphylaxia by allowing administration of a lower effective dopamine dose in accordance with the circadian cycle. In a chronic rat model using 6-OHDA-lesioning, continuous circadian i.c.v injection of A-dopamine (16h/day) over 30 days improved motor activity (i.e. ‘stepping’ and ‘cylinder’ tests and actimetry analysis) without occurrence of tachyphylaxia. This safety profile was highly favorable since A-dopamine did not induce dyskinesia or behavioral dopaminergic sensitization associated with dyskinesia as observed with peripheral L-dopa treatment (6 mg/kg twice daily). Conversely, a reduced number of apomorphine-induced rotations were observed with A-dopamine administration. Neither L-dopa or A-dopamine had supplementary deleterious effects on the remaining 10% dopaminergic neurons after ipsilateral 6-OHDA injection. Highest doses of A-dopamine may even have had a positive impact. No overall harmful effects could be attributed to body weight changes and no anatomopathological alteration of heart, liver, pancreas, spleen, kidney, spinal cord, eye, and brain were observed.

Similar to L-dopa treatment in previous clinical and preclinical studies, inconsistencies are reported in the use of dopamine, whereby high doses are described as neurotoxic but sub-toxic concentrations have neuroprotective and neurotrophic effects. We confirm that the positive impact of dopamine on dopaminergic neurons of the nigro-striatal pathways was predominantly dependent on dose, but the oxidation state of dopamine is also paramount [7].

In summary, far from being deleterious, we observe that when dopamine levels and oxidative state are harnessed it can be beneficial to neuron survival. Minimizing dopamine administrative dose, focusing delivery location and restricting the oxidative states all had a positive impact on the nigro-striatal pathways. Long-term i.c.v of A-dopamine using a circadian cycle also dramatically reduced the detrimental side effects of the current therapeutic regime of L-dopa (i.e. L-dopa related complications with dyskinesia). The study demonstrated that continuous circadian i.c.v. administration of dopamine close to the striatum is feasible, efficient and safe in mouse and rat models of PD, supporting clinical development of this strategy to be revisited in PD patients with L-dopa related complications with dyskinesia [7].
